# Identification of *Orch3*, a Locus Controlling Dominant Resistance to Autoimmune Orchitis, as Kinesin Family Member 1C

**DOI:** 10.1371/journal.pgen.1003140

**Published:** 2012-12-27

**Authors:** Roxana del Rio, Ryan D. McAllister, Nathan D. Meeker, Emma H. Wall, Jeffrey P. Bond, Vasileios C. Kyttaris, George C. Tsokos, Kenneth S. K. Tung, Cory Teuscher

**Affiliations:** 1 Department of Medicine/Immunobiology Program, University of Vermont, Burlington, Vermont, United States of America; 2 Department of Microbiology, University of Illinois, Urbana-Champaign, Illinois, United States of America; 3 Mountain States Tumor Institute, Boise, Idaho, United States of America; 4 Vermont Genetics Network Bioinformatics Core, University of Vermont, Burlington, Vermont, United States of America; 5 Division of Rheumatology, Department of Medicine, Beth Israel Deaconess Medical Center, Harvard Medical School, Boston, Massachusetts, United States of America; 6 Department of Pathology and Beirne B. Carter Center of Immunology, University of Virginia, Charlottesville, Virginia, United States of America; 7 Department of Pathology, University of Vermont, Burlington, Vermont, United States of America; The Jackson Laboratory, United States of America

## Abstract

Experimental autoimmune orchitis (EAO), the principal model of non-infectious testicular inflammatory disease, can be induced in susceptible mouse strains by immunization with autologous testicular homogenate and appropriate adjuvants. As previously established, the genome of DBA/2J mice encodes genes that are capable of conferring dominant resistance to EAO, while the genome of BALB/cByJ mice does not and they are therefore susceptible to EAO. In a genome scan, we previously identified *Orch3* as the major quantitative trait locus controlling dominant resistance to EAO and mapped it to chromosome 11. Here, by utilizing a forward genetic approach, we identified kinesin family member 1C (*Kif1c*) as a positional candidate for *Orch3* and, using a transgenic approach, demonstrated that *Kif1c* is *Orch3*. Mechanistically, we showed that the resistant *Kif1c^D2^* allele leads to a reduced antigen-specific T cell proliferative response as a consequence of decreased MHC class II expression by antigen presenting cells, and that the L^578^→P^578^ and S^1027^→P^1027^ polymorphisms distinguishing the BALB/cByJ and DBA/2J alleles, respectively, can play a role in transcriptional regulation. These findings may provide mechanistic insight into how polymorphism in other kinesins such as *KIF21B* and *KIF5A* influence susceptibility and resistance to human autoimmune diseases.

## Author Summary

Although the etiology of autoimmunity is not well known, a variety of studies have demonstrated that genetic predisposition is a major contributor to disease susceptibility and resistance. The major histocompatibility complex (MHC) is the primary genetic determinant of autoimmune disease susceptibility with multiple additional interacting loci required. However, the identification and characterization of non–MHC genes has been problematic, since most autoimmune diseases are polygenic with the individual genes exhibiting only partial or minimal penetrance. We previously identified *Orch3* (mouse chromosome 11) as the most important immune-suppressive locus controlling dominant resistance to autoimmune orchitis, the principal animal model of non-infectious testicular inflammatory/autoimmune disease. Here, using congenic mapping, we identified kinesin family member 1C (*Kif1c*) as a positional candidate for *Orch3*. Furthermore, over-expression of the *Kif1c* resistant allele in susceptible mice rendered animals autoimmune orchitis resistant, demonstrating that *Kif1c* is *Orch3*. We propose that *Kif1c* plays an immunoregulatory role by controlling the levels of MHC class II in antigen presenting cells and consequently impacting autoreactive orchitogenic T cell responses. These finding are particularly relevant since polymorphism in other kinesins such as *KIF21B* and *KIF5A* have been associated with susceptibility and resistance to human autoimmune disease.

## Introduction

Experimental autoimmune orchitis (EAO) is a model of idiopathic male infertility mediated by autoreactive T cells [1], [2]. It can be induced in mice by active immunization with mouse testicular homogenate (TH) emulsified in complete Freund's adjuvant (CFA) and *Bordetella pertussis* toxin (PTX) [3]. In genetically susceptible mice, the inflammatory lesions comprised of monocytes, macrophages, lymphocytes, neutrophils, and eosinophils are mainly found in the seminiferous tubules of the testes in association with aspermatogenesis [3]. We previously have shown that MHC class II restricted CD4^+^ T cells are the primary effectors in autoimmune orchitis [4], [5]. However, recent evidence suggests the involvement of CD8^+^ T cells during the onset and maintenance of chronic inflammation [6], [7].

Various strains of inbred mice respond differently to EAO induction, indicating that susceptibility is genetically controlled. Previously, it was shown that BALB/cByJ (CByJ) mice are highly susceptible to EAO [8] whereas DBA/2J (D2) and (CByJ×D2)F1 hybrids (CD2F1) are resistant [3], [9]. This demonstrates that resistance to EAO is inherited as a dominant phenotype in this strain combination. Additionally, resistance can be adoptively transferred to CByJ mice with CD2F1 primed splenocytes [10]. Therefore, the factors that regulate EAO resistance appear to be governed by an immune-mediated dominant negative mechanism.

Genome exclusion mapping was utilized to map the immunosuppressive genes regulating dominant resistance to EAO [10] with significant linkages to multiple loci residing on chromosomes (Chr) 1 and 11 [10]. Of these, *Orch3* on Chr11 displayed the most significant linkage and accounted for the majority of disease resistance seen in D2 mice.

In this study, congenic mapping was employed to restrict *Orch3* to a ∼1.3 Mb interval that identified *Kif1c* (kinesin family member 1c) as a positional candidate. By generating CByJ.*CD11B-Kif1c^D2^* transgenic (Tg) mice, we demonstrated that *Kif1c* underlies *Orch3*. Mechanistically, we showed that the resistant *Kif1c^D2^* allele leads to reduced antigen (Ag)-specific T cell responsiveness as a consequence of decreased MHC class II expression by myeloid cells, and that the L^578^→P^578^ and S^1027^→P^1027^ polymorphisms distinguishing the CByJ and D2 alleles, respectively, can play a role in regulating gene transcription.

## Results

### Congenic mapping of *Orch3*

**Figure 1:**
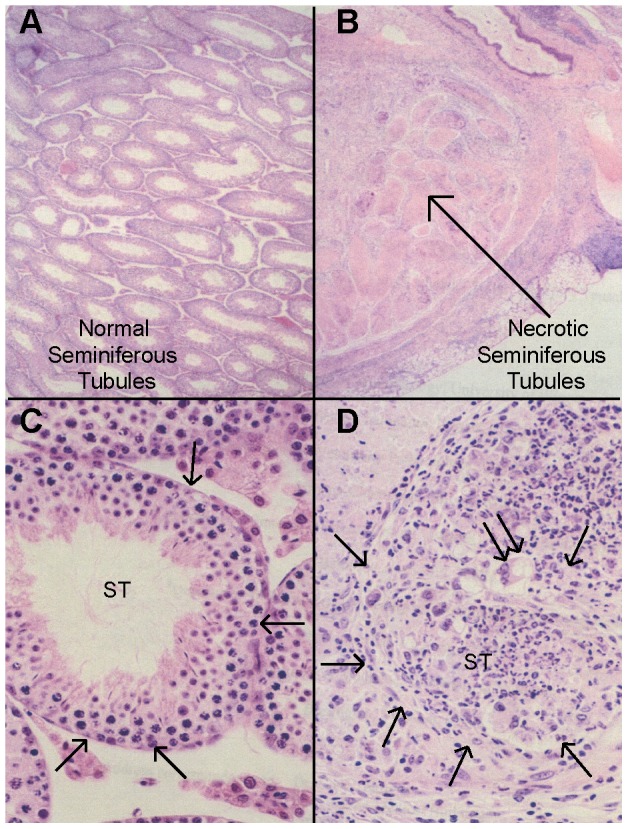
Histopathology of autoimmune orchitis. (A, C) Cross section of normal testis histology in an immunized C.D2-3 mouse: (A) Seminiferous tubules appear normal; (C) A seminiferous tubule (ST) containing normal meiotic spermatocytes and spermatids, with intact tubular boundary (arrows). (B, D) CByJ mouse with sever and diffuse orchitis: (B) All seminiferous tubules are necrotic and have lost cell nuclear staining; (D) Sever orchitis in one seminiferous tubule (arrows) that contains numerous neutrophils and occasional multinuclear giant macrophages (double arrow); the tubular boundary (arrows) is poorly defined. (H&E; A and B, ×4; C and D, ×40).

**Figure 2:**
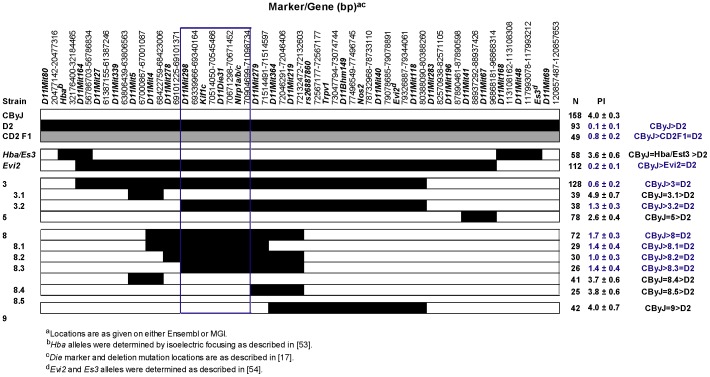
Congenic mapping places *Orch3* within the *Kif1c/Nlrp1a/b/c* interval. For convenience, *D2* alleles have been shaded. The significance of differences in severity of EAO among CByJ, CD2F1 hybrids and CD2-ISRC lines was determined using the Kruskal-Wallis test (overall *p*-value<0.0001) followed by Dunn's multiple comparison test. Region outlined in blue depicts the location of *Orch3*.

In the genome scan in which *Orch3* was identified, *D11Mit219*, *D11Mit8*, and *D11Mit118* exhibited the most significant linkage [10]. As the first step in the positional-candidate gene cloning of *Orch3*, we used marker-assisted selection to introgress the *Orch3^D2^* allele onto the susceptible CByJ background. Next, we generated overlapping interval specific recombinant congenic (ISRC) lines (Figure S1 and Figure S2) and studied them in a stepwise fashion for susceptibility and resistance to EAO (Figure 1). Importantly, since resistance to EAO is inherited as a dominant trait in CD2F1 hybrid mice [10], and the pathology indices (PI) between heterozygous and homozygous congenic lines were not significantly different (data not shown), the data were pooled for each line. Control parental CByJ mice were clearly susceptible to EAO, with an average PI of 4.0, whereas D2 and CD2F1 hybrid mice were resistant, with an average PI of 0.1 and 0.8, respectively (Figure 2). C.D2-*Es3*/*Hba*, C.D2-3.1, C.D2-5, C.D2-8.4, C.D2-8.5, and C.D2-9 mice were also susceptible with average scores of 3.6, 4.9, 2.6, 3.7, 3.8, and 4.0, respectively. In contrast, C.D2-*Evi2*, C.D2-3, C.D2-3.2, C.D2-8 and C.D2-8.1 thru -8.3 were resistant with average scores of 0.2, 0.6, 1.3, 1.7, and ≤1.4, respectively.

These data placed *Orch3* within the interval between *D11Mit298* (69339966–69340164) and NLR (nucleotide-binding domain and leucine rich repeat containing) family, pyrin domain containing 1A, B, C (*Nlrp1a/b/c*) at 70.9–71.0 Mb (70904699–71098734 bp). Importantly, this excluded transient receptor potential cation channel, subfamily V, member 1 (*Trpv1*) at 73.0 Mb (73047794–73074744) underlying *Idd4.1*, a quantitative trait loci (QTL) controlling susceptibility to type 1 diabetes in the NOD mouse [11], and inducible nitric oxide synthase (*Nos2*/*iNos*), important in inflammatory diseases including autoimmunity [12], [13], as candidate genes for *Orch3*. *Nlrp1a/b/c* is one of two highly polymorphic positional candidate loci of immunological relevance within the interval, the second gene being kinesin family member 1C (*Kif1c*). However, *Nlrp1c* could be excluded as a candidate since it is a pseudogene (www.informatics.jax.org) and *Nlrp1a* and -*b* are less likely to be relevant to *Orch3* than *Kif1c* due to discordance between EAO susceptibility and *Nlrp1a and -b* alleles among CByJ, BALB/cJ and D2 mice (www.informatics.jax.org) [14].

### CByJ.*CD11B*-*Kif1c^D2^* Tg (Tg-*Kif1c^D2^*) mice are resistant to EAO

**Figure 3:**
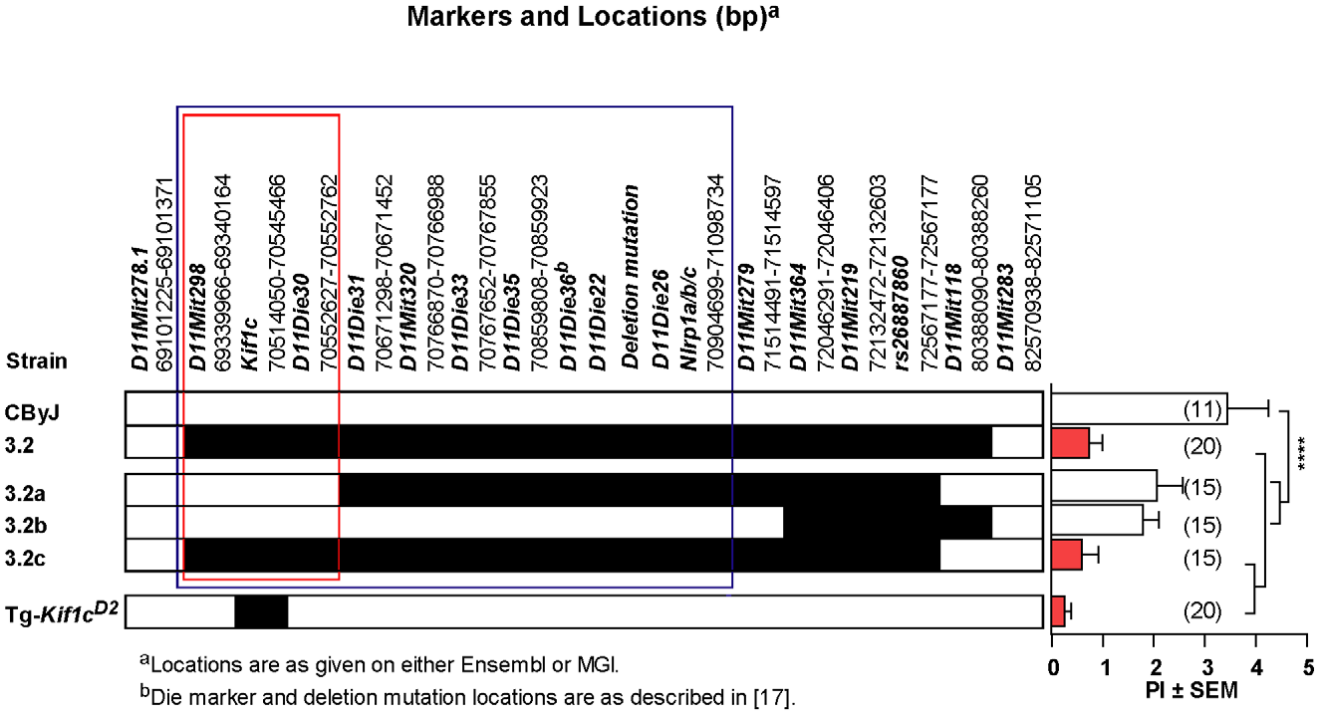
Identification of *Orch3* as *Kif1c*. (CD2-3.2×CByJ)×CByJ backcross mice were screened from recombinants using microsatellite markers spanning the *Orch3* interval. Three sub-ISRC lines were identified, fixed and homozygous progeny studied for susceptibility to EAO (D = *D2* allele; C = *CByJ* allele). The significance of differences in EAO among CByJ, CD2-3.2a, CD2-3.2b, CD2-3.2c and Tg-*Kif1c^D2^* transgenic mice was determined using the Kruskal-Wallis test (overall *p*-value<0.0001) followed by Dunn's multiple comparison test (**p<0.01). Region outlined in red reflects location of *Orch3* based on high resolution congenic mapping relative to the lower resolution mapping outlined in blue.

To confirm that *Kif1c* was the most likely candidate gene for *Orch3* and to definitively exclude *Nlrp1a/b* as a positional candidate, we generated overlapping sub-ISRC congenic lines across the C.D2-3.2 interval and studied them for susceptibility to EAO (Figure 3). Statistically significant differences in EAO susceptibility between C.D2-3.2, C.D2-3.2c and CByJ mice were observed (Figure 3, right panel). In contrast, the severity of EAO in C.D2-3.2a and C.D2-3.2b was not significantly different from that of CByJ mice. Moreover, dominant resistance co-segregated with *Orch3* as evidenced by the fact that no significant difference in the PI between homozygous and heterozygous mice was detected across all congenic lines studied (Figure 2 and Figure 3). Taken together, these data restrict *Orch3* to a ∼1.3 Mb interval distal of *D11Mit298* (69339966–69340164) and proximal of *D11Die30* (70552627–70552762) which includes *Kif1c* but not *Nlrp1a and Nlrp1b* (Figure 3, left panel), thereby excluding them as positional candidates for *Orch3*.

**Figure 4:**
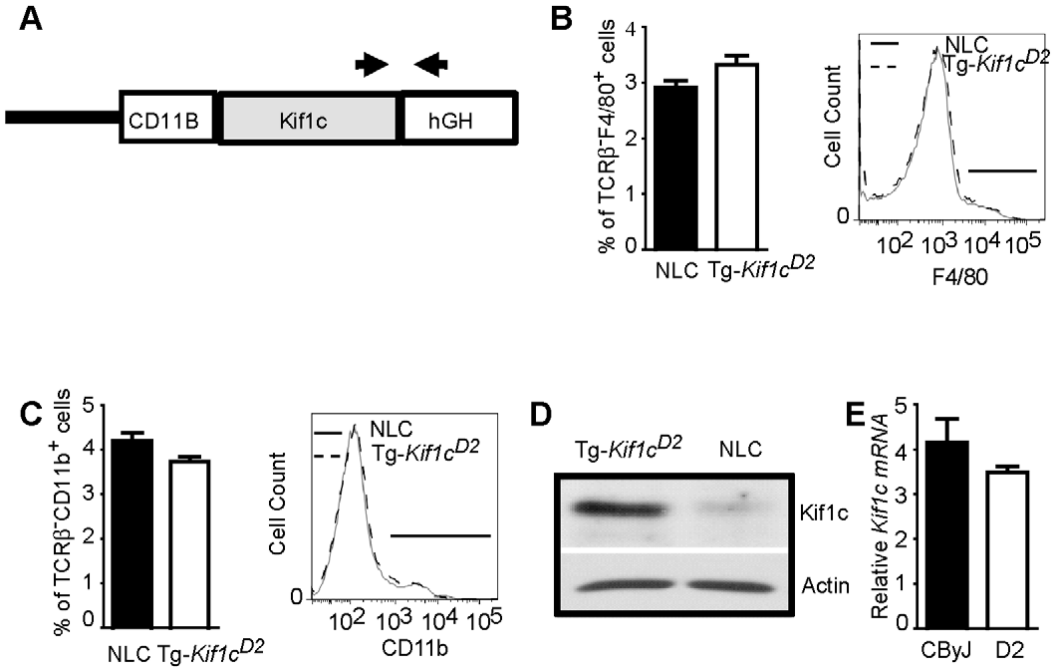
Generation of BALB/cByJ-*CD11B*-*Kif1c^D2^* transgenic (Tg-*Kif1c^D2^*) mice. (A) Schematic representation of the *Kif1c* gene used to generate the transgenic mice showing the promoter (*CD11B/ITGAM*), and the *Kif1c* gene, followed by the hGH/polyA signal sequence. Arrows indicate PCR-primers for screening. (B and C) Percentage of splenic F4/80^+^ (B) and CD11b^+^ (C) cells of Tg-*Kif1c^D2^* and NLC. The analysis was performed on gated live cells according to their FSC vs. SSC profile. Statistical significance was determined using the Mann-Whitney *U* test. Data represent the mean ± SEM of at least 5 individual mice. (D) Kif1c expression in thioglycolate-induced adherent cells by Western blotting using whole-cell extracts and the anti-Kif1c mAb. Actin was used as a loading marker. (E) mRNA expression of *Kif1c* was measured from sorted TCRβ^−^CD19^−^CD11b^+^ myeloid cells of CByJ mice and compared with TCRβ^−^CD19^−^CD11b^+^ myeloid cells of D2 mice. β2-microglobulin and GAPDH were used as an endogenous control. Data represent the mean ± SEM of two experiments (pool of 5 animals/each).

Given the role of *Kif1c* in macrophage function [15], and that kinesins have been implicated in antigen processing and presentation [16], we decided to directly test the hypothesis that *Orch3* is *Kif1c*. We generated a transgenic mouse line that selectively expressed the resistant *Kif1c^D2^* allele on the susceptible CByJ background using the human *CD11B/ITGAM* regulatory elements for macrophage/myeloid-specific expression of *Kif1c^D2^* (Figure 4A). The expression of the transgene did not affect macrophage/myeloid cell generation or homeostasis as similar percentages of splenic F4/80^+^ (Figure 4B) and CD11b^+^ cells (Figure 4C) were detected on Tg-*Kif1c^D2^* mice compared to negative littermate control (NLC) mice. In addition, no differences in the expression of CD40 or CD86 were observed between strains at baseline (data not shown). Compared to NLC, greater Kif1c protein expression was seen in thioglycolate-induced Tg-*Kif1c^D2^* cells (Figure 4D). Despite the existence of polymorphisms upstream of *Kif1c* in potential regulatory regions (http://phenome.jax.org/), we did not observe differences in *Kif1c* expression at the mRNA level between the *Kif1c^CByJ^* and *Kif1c^D2^* alleles (Figure 4E). NLC and Tg-*Kif1c^D2^* mice were studied for susceptibility to EAO. The expression of *Kif1c^D2^* in CD11b^+^ cells protected susceptible CByJ mice from developing EAO (Figure 3, right panel). This finding establishes *Kif1c* as being *Orch3*.

### Tg expression of *Kif1c^D2^* downregulates MHC class II expression and antigen presenting function of CD11b^+^ cells

**Figure 5:**
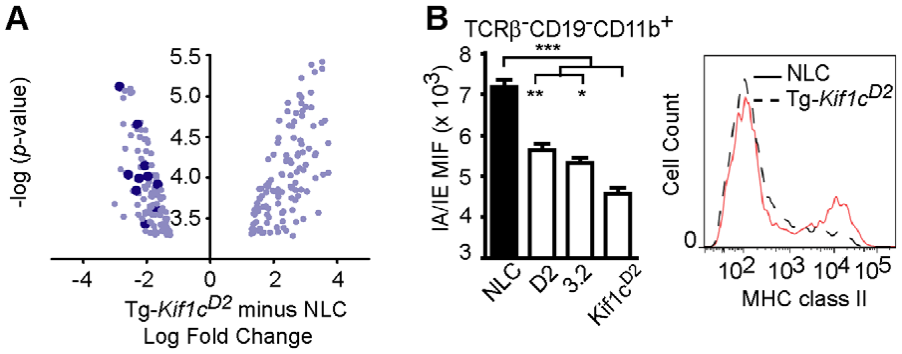
Analysis of MHC II expression on splenic CD11b^+^ myeloid cells. (A) Scatterplot of genes differentially expressed in splenic TCRβ^−^CD19^−^CD11b^+^ myeloid cells of Tg-*Kif1c^D2^* and NLC mice as determined by microarray. There were 164 genes differentially expressed (FDR≤0.05) and for each gene, the log_2_ fold change was plotted on the ordinate against the -log_10_ *p*-value, plotted on the abscissa. Each data point represents the log_2_ fold change (Tg-*Kif1c^D2^* minus NLC) for each gene. Dark blue data points indicate *H2* genes that were downregulated in splenic CD11b^+^ cells of Tg-*Kif1c^D2^* mice. (B) Flow cytometric analysis of the frequency of TCRβ^−^CD19^−^CD11b^+^-myeloid cells expressing MHC II in the spleen of Tg-*Kif1c^D2^*, D2, D.2-C3.2, and NTC. Statistical significance was determined using the Kruskal-Wallis test (overall ***p-value<0.0001) followed by Dunn's multiple comparison test (**p<0.01, *p<0.05). Data represent the mean ± SEM of at least 5 individual mice.

To better understand the mechanism of resistance to EAO conferred by *Kif1c^D2^*, microarray analyses were performed on CD11b^+^ cells from NLC and Tg-*Kif1c^D2^* mice. Using a false discovery rate (FDR) cutoff of 0.05, we determined that 164 genes were differentially expressed between NLC and Tg-*Kif1c^D2^* CD11b^+^ cells (Table S1). An analysis for functional inference using Ingenuity Pathway Analysis (Ingenuity Systems, www.ingenuity.com) revealed that T helper cell differentiation was the most significant pathway influenced by *Kif1c* (*p*<2.80 E-10; Figure S3 and Table S2). In addition, 18 of the top 20 pathways implicated a role for MHC class II, including antigen presentation. Indeed, compared to NLC CD11b^+^ cells, we observed a marked down regulation in MHC class II gene expression by Tg-*Kif1c^D2^* CD11b^+^ cells (Table S1 and Figure 5A, dark blue dots). This is consistent with the role of kinesin as the motor that drives MHC class II to the plus end of microtubules toward the cell surface [16].

To corroborate diminished class II expression, flow cytometric analysis was performed using naïve TCRβ^−^CD19^−^CD11b^+^ splenocytes. The results presented in Figure 5B show lower MHC class II expression on Tg-*Kif1c^D2^* cells compared to NLC, D2, and C.D2-3.2 mice. Despite the differences in MHC II expression, no significant difference in the proportion of total splenic CD11b^+^ cells was observed (Figure 4C). Therefore, expression of the transgene in CD11b^+^ cells negatively regulates MHC II protein levels.

**Figure 6:**
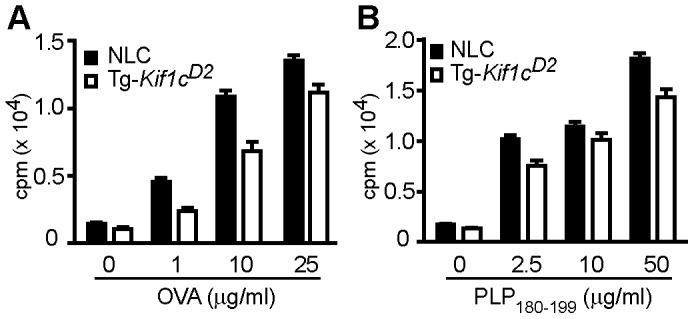
Evaluation of Ag-specific T cell stimulatory capacity of APCs. Ag-specific T cell proliferative responses were evaluated by [^3^H] thymidine incorporation. (A) OVA-specific CD4 T cells, and (B) PLP_180–199_-specific CD4 T cells from NLC mice were co-cultured with T cell-depleted/mitomycin C-treated/OVA pulsed APCs (A) and PLP_180–199_ pulsed APCs (B). Open bars are Tg-*Kif1c^D2^*-APCs, and closed bars are NLC-APCs. Each bar represents the mean cpm ± SEM of 3 independent experiments. The significance of the differences was determined by two-way ANOVA. OVA-specific response: effect of [OVA] (p<0.0001); effect of strain (p<0.0001); interaction (p = 0.08). PLP_180–199_-specific response: effect of [PLP_180–199_] (p<0.0001); effect of strain (p<0.0001); interaction (p = 0.08).

To further establish a functional role for the differential expression of MHC class II, we assessed antigen presentation by examining Ag-specific T cell proliferation. NLC and Tg-*Kif1c^D2^* mice were immunized with ovalbumin (OVA)+CFA or proteolipid protein (PLP) 180–199 peptide (PLP_180–190_)+CFA on d0 and d7. Spleen and lymph nodes (LN) were harvested at d10 and the proliferative responses evaluated. Compared to NLC antigen presenting cells (APCs), T cell proliferation in response to OVA was significantly reduced when T cells were stimulated in the presence of Tg-*Kif1c^D2^* APCs (Figure 6A). Similar results were observed for PLP_180–199_-dependent T cell responses (Figure 6B). These data show that expressing the *Kif1c^D2^* allele in CD11b^+^ cells confers resistance to EAO by modulating APC function. Taken together our data suggest that *Kif1c* coding region polymorphism controls susceptibility to autoimmune orchitis.

### Amino acid polymorphisms at residues 578 and 1027 on the C-terminal end of *Kif1c* regulate its function

**Figure 7:**
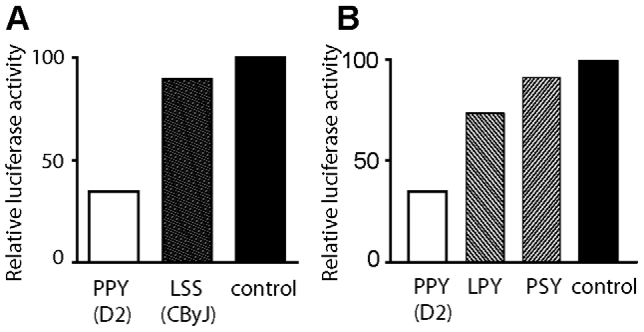
Structural polymorphisms at amino acid residues 578 and 1027 influence KIF1c function. Jurkat cells were co-transfected with a plasmid containing the (A) *Kif1c^D2^* (open bar), *Kif1c^CByJ^* (grey bar) alleles, or control plasmid (black bar), or (B) Kif1cD2 (PPY; open bar), mutant 578 (LPY; left striped bar), or mutant 1027 (PSY; right striped bar) plasmids, and *Il2* promoter luciferase reporter. Cells were stimulated for 3 hours with PMA and calcimycin, and the luciferase activity was quantified. Data are representative of two independent experiments.

*Kif1c* alleles possess amino acid substitutions at residues 578, 1027, and 1066 [17]. Four haplotypes have been identified: LSS (*Kif1c^CByJ^*), PSS, PPS, and PPY (*Kif1c^D2^*). In addition, it has been shown that the C-terminal region of KIF1c is involved in protein-protein interactions and cargo function [17]–[20]. Therefore, substitutions at 578, 1027, and/or 1066 may have a significant impact on KIF1c function. Given that KIF17b has been shown to control CREM-dependent transcription by regulating the intracellular location of the transcriptional coactivator ACT (activator of CREM in testis) [21], [22], and CREM binding to the *Il2* promoter suppresses its activity [23], we evaluated the effect of LSS *Kif1c^CByJ^* and PPY *Kif1c^D2^* alleles on *Il2* transcriptional activity as an *in vitro* assay of KIF1c allelic function. Jurkat cells were co-transfected with a plasmid containing the PPY *Kif1c^D2^* allele, the LSS *Kif1^cByJ^* allele, or an empty plasmid, and an *Il2*-promoter luciferase reporter. Cells were then activated with phorbol myristate acetate (PMA) and calcimycin, a calcium ionophore, and the luciferase activity quantified. Jurkat cells that were transfected with the plasmid containing the PPY *Kif1c^D2^* allele displayed significantly decreased luciferase activity (mean decrease 31.46±8.59%, *P* = 0.03) as compared to the plasmid containing the LSS *Kif1c^CByJ^* allele or the control plasmid (Figure 7A). These data demonstrate the functionality of the KIF1c structural polymorphism.

To further characterize the amino acid(s) responsible for the observed differences on *Il2*-promoter activity associated with the alleles, we replaced the D2-P^578^→L^578^ (LPY-KIF1c) or D2-P^1027^→S^1027^ (PSY-KIF1c). Jurkat cells were co-transfected with the plasmids containing the wild type D2 PPY-KIF1c allele, LPY-KIF1c (P^578^→L^578^) or PSY-KIF1c (P^1027^→S^1027^) mutant alleles, or a control plasmid, and *Il2*-promoter luciferase reporter. Cells were activated with PMA and calcimycin and luciferase activity was assessed. As shown in Figure 7B, LPY-KIF1c and PSY-KIF1c mutants resulted in increased *Il2*-promoter luciferase activity compared to the D2 PPY-KIF1c allele. Taken together, our data demonstrate that structural polymorphisms at position 578 and 1027 are critical for KIF1c allelic functions.

## Discussion

EAO is an organ-specific autoimmune disease that is a model of immunological male infertility [1], [2]. We previously demonstrated that genetic control of EAO is complex and involves both *H2*-linked (*Orch1*) and non-*H2*-linked (*Orch3*, *Orch4*, and *Orch5*) genes [24], [25]. The *H2*-linked immune response genes primarily control susceptibility to EAO, whereas the non-*H2*-linked genes suppress the phenotypic expression of disease associated with a susceptible *Orch1/H2* allele [9]. Here we report the identification of *Orch3* as *Kif1c* that suppresses EAO by decreasing MHC class II expression and impairing APC function. Importantly, *Kif1c* may be a shared-autoimmune gene controlling susceptibility to experimental allergic encephalomyelitis (EAE) [26]. *Eae7*, *Eae22*, and *Eae23* are linked to *Orch3* [27], and CByJ and D2 mice are susceptible and resistant to EAE, respectively [28].

With the exception of *tyrosine kinase-2* (*Tyk2*), in which a rare single nucleotide polymorphism in a well conserved APE motif within the pseudokinase domain is fully penetrant in controlling susceptibility to autoimmune diseases [29], [30], the vast majority of non-MHC autoimmune loci identified to date are QTL that exhibit only partial to minimal penetrance. This has proven to be problematic as researchers have attempted to positionally clone and characterize such genes [31]. The fact that *Orch3/Kif1c* controls a dominant negative immunoregulatory mechanism that suppresses autoimmune orchitis with a high degree of penetrance is unique. Because EAO resistance is conferred in a dominant fashion in this model, an animal must be *Orch3^CByJ^*/*Kif1c^CByJ^* homozygous to permit disease progression. By using a forward genetic approach, we have now established that *Orch3* is *Kif1c* which, in isolation, controls resistance to EAO with a remarkable degree of penetrance.

Using a transgenic approach we demonstrated that *Kif1c* is *Orch3*, and that expression of the resistant *Kif1c^D2^* allele by CD11b^+^ cells of CByJ mice confers complete protection from the development of EAO. Our data are consistent with the growing number of CD11b^+^ myeloid cell types with immunosuppressive activity [32], [33]. Indeed, resistance to autoimmune type I diabetes in NOD mice and EAE correlated with the presence of immunomodulatory CD11b^+^ myeloid cells [34]–36 and the capacity of these cells to maintain a proper T regulatory cell function [37].

Kinesin family members are involved in the activation of immune cells and inflammatory responses [38], [39], and autoimmune disease GWAS identified *KIF21B* and *KIF5A* as candidates for autoimmune disease genes [40], [41], suggesting an immunoregulatory role for kinesin family members. In addition, kinesin proteins have been identified as the major molecular motor of microtubule-based intracellular transport [42]. *Kif1c* is expressed in a variety of tissues [43] and overexpression of a dominant negative form disrupts molecular motor-dependent Golgi-to-Endoplasmic Reticulum (ER) retrograde vesicular transport [18]. It is known that *Kif1c* alleles possess amino acid substitutions at residues 578, 1027, and 1066 [17]. Here, we demonstrated that residues 578 and 1027 are functionally significant. Although the amino acid polymorphism at residue 1027 is not in an evolutionarily conserved domain [17], it is in the C-terminal region believed to participate in cargo binding. In fact, alterations of this domain have been shown to modify *in vivo* kinesin protein function [19]. Moreover, it has been shown that the C-terminal tail domain of KIF1c (amino acids 811–1090) is involved in the interaction with bicaudal-D-related protein 1 (BDRP1) and this interaction regulates secretory transport required for neurite development [20]. Therefore, the ability of KIF1c to bind and transport cargo may be altered by polymorphism in this region. However, motor-dependent Golgi-to-ER transport functions normally in *Kif1c* knockout mice [44]. Immunohistochemical staining partially co-localized KIF1c with the Golgi marker CTR433, suggesting that KIF1c may also be involved in transport around the Golgi apparatus rather than only Golgi-to-ER transport. Accordingly, Wubbolts, *et. al.* [16] showed that kinesin plays a role in the vesicular transport of MHC II-containing lysosomes from the microtubule organizing center region towards the cell surface. Here, we provide evidence that the resistant *Kif1c^D2^* allele negatively regulates the expression of MHC II proteins on APCs, since Tg-*Kif1c^D2^* CD11b^+^ cells express lower mRNA and protein levels. The reduction in MHC II expression by CD11b^+^ Tg-*Kif1c^D2^* cells was directly correlated with impaired antigen presentation as reflected by diminished Ag-specific T cell proliferative response. Whether amino acids at position 578 and 1027 on KIF1c are involved in MHC II expression is currently under investigation. Taken together, our results nevertheless provide mechanistic insight into how polymorphism in other kinesins including *KIF21B* and *KIF5A* influence human autoimmune disease susceptibility.

## Materials and Methods

### Ethics statement

Mice were housed at 25°C with 12/12-h light-dark cycles and 40–60% humidity. The experimental procedures performed in this study were under the guidelines of the Animal Care and Use Committees of the University of Vermont (Burlington, VT) and University of Illinois at Urbana-Champaign (Urbana, IL).

### Animals

BALB/cByJ (CByJ), DBA/2J (D2), and (BALB/cByJ×DBA/2J) F1 hybrid (CD2F1) mice were purchased from The Jackson Laboratory (Bar Harbor, ME). The congenic lines in this study were generated using (BALB/cAnPt×DBA/2NCr)×BALB/cAnPt backcross mice [45]. Third generation backcross mice heterozygous at *Evi2* or at *Hba* and *Es3* were selected and backcrossed for six generations to BALB/cAnPt mice and fixed by brother-sister mating to generate the C.D2-*Evi2* and C.D2-*Hba*/Es3 lines. Overlapping interval specific recombinant congenic (ISRC) lines were generated by crossing C.D2-*Evi2* mice to CByJ mice. F_2_ hybrids were genotyped using tail snip DNA and PCR with Chr11 microsatellite markers discriminating CByJ and D2 mice across the *Orch3* candidate interval. Founders were analyzed for background contamination at a density of 2–5 cM and mice carrying CByJ alleles at all background marker loci were backcrossed an additional two generations to CByJ mice. The lines were fixed by brother-sister mating to generate the C.D2-3, C.D2-5, C.D2-8, and C.D2-9 ISRC lines. Similarly, higher order resolution mapping panels of ISRC lines were generated by screening (C.D2-3×CByJ)×CByJ, (C.D2-8×CByJ)×CByJ and (C.D2-3.2×CByJ)×CByJ backcross mice for recombinants. The genealogy and complete genotypes of the C.D2 congenic mice used in this study are given in Figure S1 and Figure S2, respectively.

The CByJ.*CD11B-Kif1c^D2^* transgenic (Tg-*Kif1c^D2^*) mice were generated by microinjection with a construct containing the human *CD11B/ITGAM* promoter [46], *Kif1c^D2^* cDNA, and the *human growth hormone* (*hGH*) polyA signal [47] into C fertilized eggs at the University of Vermont Transgenic/Knockout Facility. Mice were screened for *hGH* gene by PCR using *hGH* Fwd 5′ TAG GAA GAA GCC TAT ATC CCA AAG G 3′, *hGH* Rev 5′ ACA GTC TCT CAA AGT CAG TGG GG 3′ primers. Proinsulin Fwd 5′ CTA GTT GCA GTA GTT CTC CAG 3′ and proinsulin Rev 5′ CCT GCC TAT CTT TCA GGT C 3′ primers were used as internal control.

### PCR–based restriction fragment length polymorphism (RFLP)

Genomic DNA was PCR-amplified using standard conditions and the following primers designed around a polymorphism in *Nlrp1a*: 5′-GGGCACATGGATTCAGAGAT-3′; 5′-AGAGACCCCACCCAACTTC-3′. 10 µl of PCR reaction was digested using 5 units of *ApaLI* in 50 µl of 1× NEBuffer 4 (New England BioLabs, Inc., Ipswich, MA) for 1 hour at 37°C. Resulting fragments were electrophoresed in 2% agarose gels and visualized by ethidium bromide.

### EAO induction

Six-12 week old mice were immunized as previously described [9] with 10 mg of TH plus CFA (Sigma-Aldrich, St. Louis, MO) supplemented with 200 µg of *Mycobacterium tuberculosis* H37Ra (Difco Laboratories, Detroit, MI) in conjunction with PTX (List Biological Laboratories Inc., Campbell, CA). EAO was evaluated at 25–30 days post-injection. The testes were processed for histological examination as previously described [9]. Histopathologic analysis was carried out in a double-blind manner with each testis being scored individually on a PI from 0–10 as described previously [9]. The overall score for each animal was calculated as the average of both testes with the strain means representing the average of the averages.

### Cell preparation and Western blotting

Spleens were collected from CByJ and Tg-*Kif1c^D2^* mice, and single cell suspensions were prepared by passing the cells through a 50 µm nylon mesh (Small parts Inc, Miami Lakes, FL). Erythrocytes were lysed using complete Geyes solution, washed two times and plated to obtain adherent cells. Adherent cells were removed by treating with 0.025% Trypsin-EDTA (Invitrogen, Carlsbad, California), washed three times and pelleted. Whole-cell lysates were prepared in Triton lysis buffer and equal amounts of protein were then separated via SDS-PAGE and transferred to nitrocellulose membranes as described previously [48]. Primary antibodies used for Western blot include anti-Kif1c and anti-Actin (Santa Cruz Biotechnology Inc., Santa Cruz, CA). Bound antibody was visualized by peroxidase-conjugated secondary antibody and detected by chemiluminescence (Kirkegaard and Perry Laboratories, Gaithersburg, MD).

### FACS sorting and flow cytometric analysis

NLC and Tg-*Kif1c^D2^* myeloid cells from erythrocyte-free spleens were first enriched by negative selection (using magnetic beads, Qiagen, Hilden, Germany) to deplete cells expressing CD8, CD4, and IgM. For FACS isolation, negatively selected enriched-myeloid cells were stained with anti-CD11b–APCCy7 (BD Pharmingen. Franklin Lakes, NJ), anti-CD11c-PECy5.5 (Invitrogen, Camarillo, CA), anti-TCRβ–FITC, and anti-IA/IE-PE (eBioscience, San Diego, CA), and sorted on a FACSAria (BD Biosciences, San Jose, CA) by gating in the TCRβ^−^IA/IE^+^CD11c^−^CD11b^+^ myeloid cell population. Antibodies against B220 and CD19 (eBioscience) were also used for flow cytometry.

### Microarray analysis

Total RNA was extracted and purified from TCRβ^−^IA/IE^+^CD11c^−^CD11b^+^ myeloid cells from naïve NLC and Tg-*Kif1c^D2^* mice (n = 6 to 10 mice/strain) using RNeasy isolation reagent (Qiagen Inc.). Purified RNA was quantified using a Nanodrop ND1000™ spectrophotometer (Thermo Scientific, Wilmington, DE) and quality was assessed using an Agilent 2100 bioanalyzer (Agilent Technologies, Palo Alto, California). The RNA integrity number of all samples was greater than 8. For microarray analysis, two RNA pools were created so that each pool contained RNA from 3 to 5 mice, and two arrays per strain were analyzed.

RNA amplification and microarray analysis was performed at UVM Microarray Core Facility using previously described protocols [49]. Briefly, 2 µg of total RNA from each pooled sample were reverse transcribed to the single stranded cDNA using T7-oligo(dT) primer. T4 DNA polymerase was used to synthesize double-stranded cDNA, which served as a template for *in vitro* transcription using T7 RNA polymerase to produce biotinylated cRNA. The biotinylated cRNAs were fragmented into 50- to 200-base fragments and then hybridized to GeneChip Mouse Genome 430A 2.0 Arrays for 16 h at 45°C in a rotating Affymetrix GeneChip Hybridization Oven 320. After hybridization, arrays were washed and stained with streptavidin-phycoerythrin on an automated Affymetrix GeneChip Fluidic Station F450. The arrays were scanned with an Affymetrix GeneChip Scanner 2700 and the images quantified using Affymetrix GeneChip Operating Software.

The signal intensity for each probe on each chip was calculated from scanned images using GeneChip Operating Software (Affymetrix), and signal intensities were analyzed using BioConductor (http://www.bioconductor.org). Probe intensities were background corrected, normalized, and summarized using the Robust Multichip Average method described by Speed and coworkers [50], [51]. An alternative normalization method based on reference genes did not significantly change the results. The FDR for differential expression between NLC and Tg-*Kif1c^D2^* for each individual gene was calculated using the method of Benjamini and Hochberg [52]. Gene expression data were analyzed using a threshold of FDR≤0.05 to identify differentially expressed genes.

### T cell stimulatory capacity of antigen presenting cells (APCs)

NLC and Tg-*Kif1c^D2^* mice were immunized at d0 and d7 s.c. in the posterior right and left flank and the scruff of the neck with a sonicated PBS/oil emulsion containing 20 µg of OVA, faction V (Sigma-Aldrich, St. Louis, MO), or 100 µg of PLP_180–199_ in CFA supplemented with 200 µg of *Mycobacterium tuberculosis* H37Ra. Spleens and LN were harvested on d10. APCs from erythrocyte-free spleens were obtained by anti-CD4/anti-CD8 complement depletion and treated with mitomycin C (25 µg/ml; Sigma-Aldrich). Responder CD4 T cells from LN and spleens were isolated by negative selection as previously described [48]. Single cell suspensions of OVA- or PLP_180–199_-APCs (2×10^5^ cells/well) and Ag-specific responder CD4 T cell (1×10^5^ cells/well) suspensions were prepared in RPMI 1640 (5% FBS), and plated on standard 96-well U-bottom tissue culture plates. Cells were stimulated with 1, 10, and 25 µg/ml of OVA or 2.5, 10, and 50 µg/ml of PLP_180–199_ for 72 h at 37°C. During the last 18 h of culture, 1 µCi of [^3^H] thymidine (PerkinElmer, Santa Clara, CA) was added. Cells were harvested onto glass fiber filters and thymidine uptake was determined with a liquid scintillation counter.

### Preparation of Jurkat cells, transfection, stimulation, and luciferase assays

Jurkat cells were cultured in RPMI containing 10% FBS without stimulation for 24 hours at a concentration of 1×10^6^ cells/ml. Plasmids encoding *Kif1c^D2^*, *Kif1c^CByJ^* alleles, LPY-KIF1c and PSY-KIF1c mutants, corresponding empty vector (pcDNA, Invitrogen, Carlsbad, CA), *Il2* promoter (−575 to +57 base pairs) luciferase reporter, and control pGL2 luciferase reporter (Promega, Madison, WI) were used for transfection. Five micrograms of each plasmid were used for the transfection of approximately 5×10^6^ Jurkat cells by electroporation at 250 mV and 900 µF in 250 µl of RPMI with a BioRad electroporator (BioRad, Hercules, CA). Cells were subsequently cultured in RPMI and 10% FBS for 24 hours and then stimulated with PMA (10 ng/ml) and calcium ionophore calcimycin (0.5 µg/ml) for 3 hours. Cell lysates were prepared and supernatants collected to quantified luciferase activity (Promega, Madison, WI). The luminescence was measured immediately using a luminometer (Sunnyvale, CA). The transfection efficiency was compared between the samples by co-transfecting a plasmid encoding β-galactosidase. The luciferase activity was normalized using the β-galactosidase value.

### Mutagenesis

Point mutations were introduced in the plasmid encoding the *Kif1c* allele from the D2 mouse using the QuikChange Site-Directed mutagenesis kit (Stratagene, USA). Briefly the plasmid was denatured and then annealed with the appropriate mutagenic primer that contained the desired mutation. Using *Pfu* DNA polymerase, new mutagenized strands were created. The parental DNA template was digested with *DpnI* and the new mutated plasmid was used to transform *E. coli*. The plasmid DNA was extracted using the Qiagen Maxi-Prep kit (Qiagen, Valencia, CA). The primers used for mutagenesis of the nucleotide at position 1033 (amino acid 578) of the D2 allele were: forward: 5′-GCTCGTGACGGAGCTGCTGGTGCTGAAGTC-3′; reverse: 5′-GACTTCAGCACCAGCAGCTCCGTCACGAGC- 3′; and for the nucleotide at position 3079 (amino acid 1027):

Forward: 5′CGAAGACCCCACCGTTCTCGCAGGAATTCCC-3′, and

Reverse: 5′GGGAATTCCTGCGAGAACGGTGGGGTCTTCG-3′.

## Supporting Information

Figure S1 Genealogy of the congenic and interval specific congenic lines used in this study. Third backcross generation (BALB/cAnPt×DBA/2NCr)×BALB/cAnPt mice heterozygous at *Evi2* or at *Hba* and *Es3* were selected and backcrossed for six generations to BALB/cAnPt mice. Homozygous lines C.D2-*Evi2* and C.D2-*Hba*/Es3 were fixed by brother-sister mating. Overlapping interval specific recombinant congenic (ISRC) lines were generated by crossing C.D2-*Evi2* mice to CByJ mice. F_2_ hybrids were genotyped using tail snip DNA and PCR with Chr11 microsatellite markers discriminating CByJ and D2 mice across the *Orch3* candidate interval [10]. Founders were analyzed for background contamination and mice carrying CByJ alleles at all background marker loci were backcrossed an additional two generations to CByJ mice. Homozygous C.D2-3, C.D2-5, C.D2-8, and C.D2-9 ISRC lines were fixed by brother-sister mating. Similarly, higher order resolution mapping panels of ISRC lines were generated by screening (C.D2-3×CByJ)×CByJ, (C.D2-8×CByJ)×CByJ and (C.D2-3.2×CByJ)×CByJ backcross mice for recombinants.
(PDF)

Figure S2 Genotypes of congenic and interval specific congenic lines used in this study. Microsatellite and SNP based genotyping was done using tail snip DNA and PCR [10]. ^a^Locations are as given on either Ensembl or MGI. ^b^ *Hba* alleles were determined by isoelectric focusing as described in [53]. ^c^ *Die* marker and deletion mutation locations are as described in [54]. ^d^ *Evi2* and *Es3* alleles were determined as described in [55].
(PDF)

Figure S3 Illustration of the potential effect of altered MHC Class II expression on T helper (TH) cell differentiation. (Figure generated using Ingenuity Pathway Analysis, Ingenuity Systems. Green = expression decreased in Tg-*Kif1c^D2^* relative to NLC).
(PDF)

Table S1 Genes differentially expressed between NLC and Tg-*Kif1c^D2^* CD11b^+^ cells. TCRβ^−^IA/IE^+^CD11c^−^CD11b^+^ myeloid cells were harvested from naïve NLC and Tg-*Kif1c^D2^* mice and differential gene expression was detected using Affymetrix GeneChip Mouse Genome 430A 2.0 Arrays. ^1^Chr = chromosome. ^2^logFC = log_2_ signed fold change.
(PDF)

Table S2 Transgenic expression of *Kif1c^D2^* on CD11b^+^ cells influences pathways involving MHC Class II (genes in bold). TCRβ^−^IA/IE^+^CD11c^−^CD11b^+^ myeloid cells were harvested from naïve NLC and Tg-*Kif1c^D2^* mice and differential gene expression was detected using Affymetrix GeneChip Mouse Genome 430A 2.0 Arrays. ^1^Pathway analysis was conducted using Ingenuity Pathway Analysis software (Ingenuity Systems, www.ingenuity.com). To ensure biological relevance, cell type was restricted to B-cells, dendritic cells, and macrophages.
(PDF)
