# Supplementary figures and images for: Identification of Orch3, a Locus Controlling Dominant Resistance to Autoimmune Orchitis, as Kinesin Family Member 1C
Source: PLoS Genet. 2012 Dec 27;8(12):e1003140. doi: 10.1371/journal.pgen.1003140 (PMC3531464; doi:10.1371/journal.pgen.1003140)

## Genealogy of C.D2 Congenic Lines

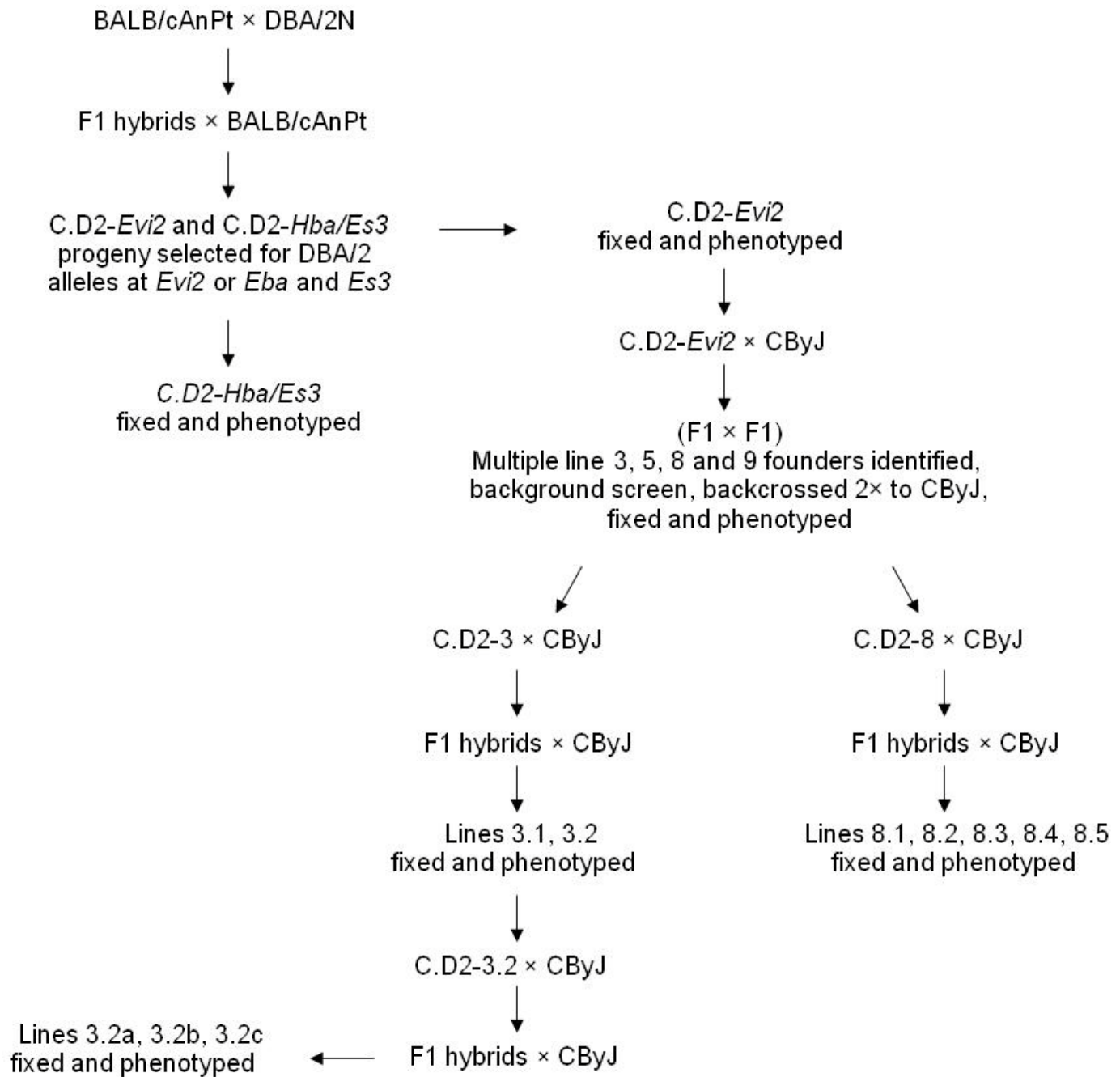

Supplement: Figure S1 — Genealogy of the congenic and interval specific congenic lines used in this study. Third backcross generation (BALB/cAnPt×DBA/2NCr)×BALB/cAnPt mice heterozygous at Evi2 or at Hba and Es3 were selected and backcrossed for six generations to BALB/cAnPt mice. Homozygous lines C.D2-Evi2 and C.D2-Hba/Es3 were fixed by brother-sister mating. Overlapping interval specific recombinant congenic (ISRC) lines were generated by crossing C.D2-Evi2 mice to CByJ mice. F2 hybrids were genotyped using tail snip DNA and PCR with Chr11 microsatellite markers discriminating CByJ and D2 mice across the Orch3 candidate interval [10]. Founders were analyzed for background contamination and mice carrying CByJ alleles at all background marker loci were backcrossed an additional two generations to CByJ mice. Homozygous C.D2-3, C.D2-5, C.D2-8, and C.D2-9 ISRC lines were fixed by brother-sister mating. Similarly, higher order resolution mapping panels of ISRC lines were generated by screening (C.D2-3×CByJ)×CByJ, (C.D2-8×CByJ)×CByJ and (C.D2-3.2×CByJ)×CByJ backcross mice for recombinants. (PDF) [file pgen.1003140.s001.pdf]

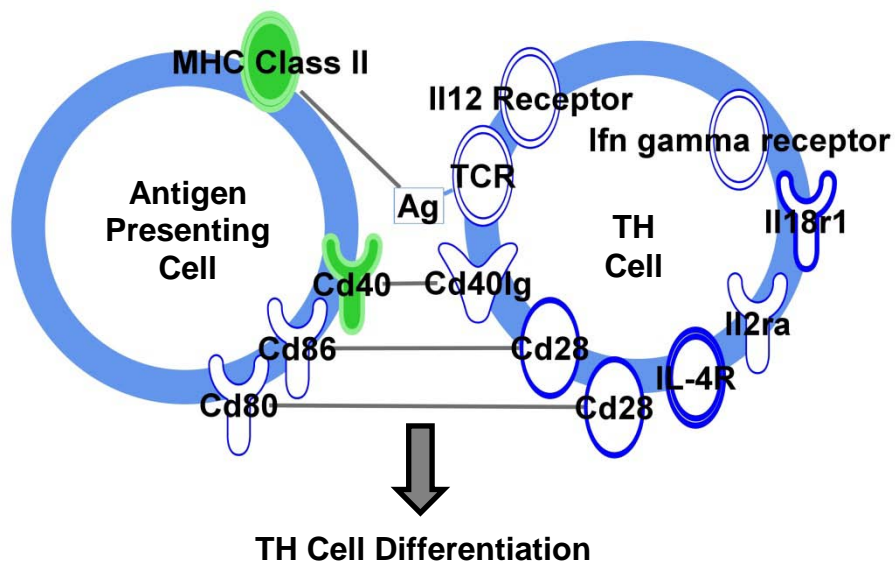

Supplement: Figure S3 — Illustration of the potential effect of altered MHC Class II expression on T helper (TH) cell differentiation. (Figure generated using Ingenuity Pathway Analysis, Ingenuity Systems. Green = expression decreased in Tg-Kif1cD2 relative to NLC). (PDF) [file pgen.1003140.s003.pdf]
