# Supplementary material for: Identification of Orch3, a Locus Controlling Dominant Resistance to Autoimmune Orchitis, as Kinesin Family Member 1C
Source: PLoS Genet. 2012 Dec 27;8(12):e1003140. doi: 10.1371/journal.pgen.1003140 (PMC3531464; doi:10.1371/journal.pgen.1003140)
Supplement: Figure S2 — Genotypes of congenic and interval specific congenic lines used in this study. Microsatellite and SNP based genotyping was done using tail snip DNA and PCR [10]. aLocations are as given on either Ensembl or MGI. b Hba alleles were determined by isoelectric focusing as described in [53]. c Die marker and deletion mutation locations are as described in [54]. d Evi2 and Es3 alleles were determined as described in [55]. (PDF) [file pgen.1003140.s002.pdf]

Marker/Gene	Location (bp)[a]	Strain CByJ	D2	C.D2-*Hba/Es3*	C.D2-*Evi2*	C.D2-3	C.D2-3.1	C.D2-3.2	C.D2-3.2a	C.D2-3.2b	C.D2-3.2c	C.D2-5	C.D2-8	C.D2-8.1	C.D2-8.2	C.D2-8.3	C.D2-8.4	C.D2-8.5	C.D2-8.6	C.D2-9
D11Mit80	20477142-20477316	C	D	C	C	C	C	C	C	C	C	C	C	C	C	C	C	C	C	C
Hba [b]	32176400-32184465	C	D	D	C	C	C	C	C	C	C	C	C	C	C	C	C	C	C	C
D11Mit135	33566590-33566736	C	D	D	C	C	C	C	C	C	C	C	C	C	C	C	C	C	C	C
D11Mit269	42571003-42571127	C	D	D	C	C	C	C	C	C	C	C	C	C	C	C	C	C	C	C
D11Mit296	42695301-42695416	C	D	D	C	C	C	C	C	C	C	C	C	C	C	C	C	C	C	C
D11Mit235	44187191-44187370	C	D	D	C	C	C	C	C	C	C	C	C	C	C	C	C	C	C	C
D11Mit237	44187223-44187370	C	D	D	C	C	C	C	C	C	C	C	C	C	C	C	C	C	C	C
D11Mit20	44552713-44552826	C	D	D	C	C	C	C	C	C	C	C	C	C	C	C	C	C	C	C
D11Mit271	45631256-45631375	C	D	D	C	C	C	C	C	C	C	C	C	C	C	C	C	C	C	C
D11Mit314	51375154-51375277	C	D	D	C	C	C	C	C	C	C	C	C	C	C	C	C	C	C	C
D11Mit240	53042439-53042588	C	D	D	C	C	C	C	C	C	C	C	C	C	C	C	C	C	C	C
D11Mit86	54003617-54003740	C	D	D	C	C	C	C	C	C	C	C	C	C	C	C	C	C	C	C
D11Mit140	54016997-54017124	C	D	D	C	C	C	C	C	C	C	C	C	C	C	C	C	C	C	C
D11Mit131	55969016-55969165	C	D	D	C	C	C	C	C	C	C	C	C	C	C	C	C	C	C	C
D11Mit274	56555162-56555275	C	D	D	C	C	C	C	C	C	C	C	C	C	C	C	C	C	C	C
D11Mit164	56786703-56786834	C	D	D	D	D	C	C	C	C	C	C	C	C	C	C	C	C	C	C
D11Mit275	61387155-61387246	C	D	C	D	D	C	C	C	C	C	C	C	C	C	C	C	C	C	C
D11Mit157	61805969-61806113	C	D	C	D	D	C	C	C	C	C	C	C	C	C	C	C	C	C	C
D11Mit261	61964703-61964810	C	D	C	D	D	C	C	C	C	C	C	C	C	C	C	C	C	C	C
D11Mit318	62019885-62019985	C	D	C	D	D	C	C	C	C	C	C	C	C	C	C	C	C	C	C
D11Mit88	62964789-62965039	C	D	C	D	D	C	C	C	C	C	C	C	C	C	C	C	C	C	C
D11Mit156	63323910-63324021	C	D	C	D	D	C	C	C	C	C	C	C	C	C	C	C	C	C	C
D11Mit339	63806439-63806563	C	D	C	D	D	C	C	C	C	C	C	C	C	C	C	C	C	C	C
D11Mit5	67000867-67001087	C	D	C	D	D	D	C	C	C	C	C	C	C	C	C	D	C	D	C
D11Mit4	68422759-68423006	C	D	C	D	D	D	C	C	C	C	C	D	D	C	C	D	C	D	C
D11Mit278.1	69101225-69101371	C	D	C	D	D	C	C	C	C	C	C	D	D	D	C	C	C	D	C
D11Mit298	69339966-69340164	C	D	C	D	D	C	D	C	C	D	C	D	D	D	D	C	C	C	C
D11Mit29	69607483-69607623	C	D	C	D	D	C	D	C	C	D	C	D	D	D	D	C	C	C	C
D11Mit30	69607486-69607663	C	D	C	D	D	C	D	C	C	D	C	D	D	D	D	C	C	C	C
D11Mit15	69761978-69762125	C	D	C	D	D	C	D	C	C	D	C	D	D	D	D	C	C	C	C
D11Mit243	69854660-69854792	C	D	C	D	D	C	D	C	C	D	C	D	D	D	D	C	C	C	C
D11Mit90	70313264-70313413	C	D	C	D	D	C	D	C	C	D	C	D	D	D	D	C	C	C	C
Kif1c	70514050-70545466	C	D	C	D	D	C	D	C	C	D	C	D	D	D	D	C	C		C
D11Die30 [c]	70552627-70552762	C	D	C	D	D	C	D	C	C	D	C	D	D	D	D	C	C	C	C
D11Die31	70671298-70671452	C	D	C	D	D	C	D	D	C	D	C	D	D	D	D	C	C	C	C
D11Mit320	70766870-70766988	C	D	C	D	D	C	D	D	C	D	C	D	D	D	D	C	C	C	C
D11Die33	70767652-70767855	C	D	C	D	D	C	D	D	C	D	C	D	D	D	D	C	C	C	C
D11Die34		C	D	C	D	D	C	D	D	C	D	C	D	D	D	D	C	C	C	C
D11Die35	70859808-70859923	C	D	C	D	D	C	D	D	C	D	C	D	D	D	D	C	C	C	C
D11Die36		C	D	C	D	D	C	D	D	C	D	C	D	D	D	D	C	C	C	C
D11Die22																				
Deletion mutation		C	D	C	D	D	C	D	D	C	D	C	D	D	D	D	C	C	C	C
D11Die26																				
Nlrp1a/b/c	70904699-71098734	C	D	C	D	D	C	D	D	C	D	C	D	D	D	D	C	C	C	C
D11Die37		C	D	C	D	D	C	D	D	C	D	C	D	D	D	D	C	D	C	C
D11Die38		C	D	C	D	D	C	D	D	C	D	C	D	D	D	D	C	D	C	C
D11Mit279	71514491-71514597	C	D	C	D	D	C	D	D	C	D	C	D	D	D	D	C	D	C	C
D11Mit364	72046291-72046406	C	D	C	D	D	C	D	D	D	D	C	D	C	D	D	C	D	C	D
D11Mit219	72132472-72132603	C	D	C	D	D	C	D	D	D	D	C	D	C	D	D	C	D	C	D
rs26887860	72567177-72567177	C	D	C	D	D	C	D	D	D	D	C	C	C	C	C	C	C	C	D

		Strain																		
**Marker/Gene**	**Location (bp)[a]**	**CByJ**	**D2**	**C.D2-*Hba/Es3***	**C.D2-*Evi2***	**C.D2-3**	**C.D2-3.1**	**C.D2-3.2**	**C.D2-3.2a**	**C.D2-3.2b**	**C.D2-3.2c**	**C.D2-5**	**C.D2-8**	**C.D2-8.1**	**C.D2-8.2**	**C.D2-8.3**	**C.D2-8.4**	**C.D2-8.5**	**C.D2-8.6**	**C.D2-9**
Trpv1	73047794-73074744	C	D	C	D	D	C	D	D	D	D	C	C	C	C	C	C	C	C	D
D11Bhm149	77496549-77496745	C	D	C	D	D	C	D	C	D	C	C	C	C	C	C	C	C	C	D
D11Bhm154	78285771-78285892	C	D	C	D	D	C	D	C	D	C	C	C	C	C	C	C	C	C	D
D11Mit144/Nos2	78732968-78733110	C	D	C	D	D	C	D	C	D	C	C	C	C	C	C	C	C	C	D
D11Bhm163	78748077-78748176	C	D	C	D	D	C	D	C	D	C	C	C	C	C	C	C	C	C	D
D11Mit34	79078681-79078829	C	D	C	D	D	C	D	C	D	C	C	C	C	C	C	C	C	C	D
D11Mit40	79078685-79078891	C	D	C	D	D	C	D	C	D	C	C	C	C	C	C	C	C	C	D
D11Mit94	79088251-79088376	C	D	C	D	D	C	D	C	D	C	C	C	C	C	C	C	C	C	D
Evi2 [d]	79326887-79344061	C	D	C	D	D	C	D	C	D	C	C	C	C	C	C	C	C	C	D
D11Mit262	79579406-79579508	C	D	C	D	D	C	D	C	D	C	C	C	C	C	C	C	C	C	D
D11Mit8	79736435-79736588	C	D	C	D	D	C	D	C	D	C	C	C	C	C	C	C	C	C	D
D11Mit118	80388090-80388260	C	D	C	D	D	C	D	C	D	C	C	C	C	C	C	C	C	C	D
D11Mit283	82570938-82571105	C	D	C	D	C	C	C	C	C	C	C	C	C	C	C	C	C	C	C
D11Mit120	83403911-83404032	C	D	C	D	C	C	C	C	C	C	C	C	C	C	C	C	C	C	C
D11Mit97	83416806-83417067	C	D	C	D	C	C	C	C	C	C	C	C	C	C	C	C	C	C	C
D11Mit326	83563753-83563847	C	D	C	D	C	C	C	C	C	C	C	C	C	C	C	C	C	C	C
D11Mit36	83656096-83656328	C	D	C	D	C	C	C	C	C	C	C	C	C	C	C	C	C	C	C
D11Mit281	84438051-84438172	C	D	C	D	C	C	C	C	C	C	C	C	C	C	C	C	C	C	C
D11Mit195	84654000-84654138	C	D	C	D	C	C	C	C	C	C	C	C	C	C	C	C	C	C	C
D11Mit121	84783528-84783647	C	D	C	D	C	C	C	C	C	C	C	C	C	C	C	C	C	C	C
D11Mit159	84967740-84967861	C	D	C	D	C	C	C	C	C	C	C	C	C	C	C	C	C	C	C
D11Mit165	85796804-85796945	C	D	C	D	C	C	C	C	C	C	C	C	C	C	C	C	C	C	C
D11Mit211	86262879-86263016	C	D	C	D	C	C	C	C	C	C	C	C	C	C	C	C	C	C	C
D11Mit282	86718716-86718841	C	D	C	D	C	C	C	C	C	C	C	C	C	C	C	C	C	C	C
D11Mit325	86874328-86874451	C	D	C	D	C	C	C	C	C	C	C	C	C	C	C	C	C	C	C
D11Mit196	87890461-87890598	C	D	C	D	C	C	C	C	C	C	C	C	C	C	C	C	C	C	C
D11Mit41	88937292-88937426	C	D	C	D	C	C	C	C	C	C	D	C	C	C	C	C	C	C	C
D11Mit67	96868181-96868314	C	D	C	D	C	C	C	C	C	C	D	C	C	C	C	C	C	C	C
D11Mit168	113108162-113108308	C	D	D	C	C	C	C	C	C	C	C	C	C	C	C	C	C	C	C
D11Mit48	117993078-117993212	C	D	D	C	C	C	C	C	C	C	C	C	C	C	C	C	C	C	C
Es3 [d]		C	D	D	C	C	C	C	C	C	C	C	C	C	C	C	C	C	C	C
D11Mit69	120857487-120857653	C	D	C	C	C	C	C	C	C	C	C	C	C	C	C	C	C	C	C

[a]Locations are as given on either Ensembl or MGI.

[b]*Hba* alleles were determined by isoelectric focusing as described in [53].

[c]*Die* marker and deletion mutation locations are as described in [17].

[d]*Evi2* and *Es3* alleles were determined as described in [54].
