# Supplementary material for: Identification of Orch3, a Locus Controlling Dominant Resistance to Autoimmune Orchitis, as Kinesin Family Member 1C
Source: PLoS Genet. 2012 Dec 27;8(12):e1003140. doi: 10.1371/journal.pgen.1003140 (PMC3531464; doi:10.1371/journal.pgen.1003140)
Supplement: Table S1 — Genes differentially expressed between NLC and Tg-Kif1cD2 CD11b+ cells. TCRβ−IA/IE+CD11c−CD11b+ myeloid cells were harvested from naïve NLC and Tg-Kif1cD2 mice and differential gene expression was detected using Affymetrix GeneChip Mouse Genome 430A 2.0 Arrays. 1Chr = chromosome. 2logFC = log2 signed fold change. (PDF) [file pgen.1003140.s004.pdf]

Gene Symbol	Gene Name	Chr[1]	Tg-Kif1c vs NLC logFC[2]	Tg-Kif1c vs NLC *P*-Value	Probe set ID
Abcc5	ATP-binding cassette, sub-family C (CFTR/MRP), member 5	16	-1.42	3.2E-04	1435684_at
Aif1	allograft inflammatory factor 1	17	-2.73	3.0E-04	1418204_s_at
Arap2	ArfGAP with RhoGAP domain, ankyrin repeat and PH domain 2	5	2.71	3.5E-04	1452291_at
B3gnt5	UDP-GlcNAc:betaGal beta-1,3-N-acetylglucosaminyltransferase 5	16	1.47	2.3E-04	1420994_at
Bace1	beta-site APP cleaving enzyme 1	9	-1.40	3.5E-04	1455826_a_at
Bcl11a	B-cell CLL/lymphoma 11A (zinc finger protein)	11	-1.90	3.5E-04	1419406_a_at
Bfar	bifunctional apoptosis regulator	16	-2.02	4.7E-05	1426490_at
Bhlhe40	basic helix-loop-helix family, member e40	6	3.56	9.2E-05	1418025_at
Btg3	B-cell translocation gene 3	17	2.82	7.7E-05	1449007_at
Camkk2	calcium/calmodulin-dependent protein kinase kinase 2, beta	5	-1.89	2.7E-04	1424474_a_at
Car2	carbonic anhydrase 2	3	2.87	1.2E-05	1448752_at
Cbfa2t3	core-binding factor, runt domain, alpha subunit 2, translocated to, 3	8	-1.36	3.2E-04	1438705_at
Ccdc109b	coiled-coil domain containing 109B	3	-1.73	9.6E-05	1418778_at
Ccl3	chemokine (C-C motif) ligand 3	11	2.27	9.2E-05	1419561_at
Ccl4	chemokine (C-C motif) ligand 4	11	3.33	5.1E-05	1421578_at
Ccl5	chemokine (C-C motif) ligand 5	11	2.93	3.2E-04	1418126_at
Ccl6	chemokine (C-C motif) ligand 6	11	-1.86	5.1E-05	1420249_s_at
Ccl9	chemokine (C-C motif) ligand 9	11	-1.48	1.7E-04	1417936_at
Ccnd2	cyclin D2	6	2.27	2.7E-04	1434745_at
Ccr2	chemokine (C-C motif) receptor 2	9	-1.69	1.0E-04	1421188_at
Ccr5	chemokine (C-C motif) receptor 5	9	1.63	1.7E-04	1424727_at
Cd2	CD2 antigen	3	3.32	1.0E-04	1418770_at
Cd28	CD28 antigen	1	2.25	8.9E-05	1437025_at
Cd40	CD40 antigen	2	-1.26	5.1E-04	1449473_s_at
Cd68	CD68 antigen	11	-1.99	3.4E-05	1449164_at
Cd74	CD74 antigen	18	-2.58	9.2E-05	1425519_a_at
Cd83	CD83 antigen	13	2.10	1.5E-04	1416111_at
Cd93	CD93 antigen	2	-1.65	1.2E-04	1419589_at
Cebpa	CCAAT/enhancer binding protein (C/EBP), alpha	7	-2.08	3.4E-05	1418982_at
Celsr3	cadherin, EGF LAG seven-pass G-type receptor 3	9	-1.97	9.6E-05	1425067_at
Clip4	CAP-GLY domain containing linker protein family, member 4	17	2.56	1.3E-04	1427278_at
Crem	cAMP responsive element modulator	18	2.91	1.5E-05	1449037_at
Csf1r	colony stimulating factor 1 receptor	18	-2.07	1.3E-04	1419872_at
Cst7	cystatin F (leukocystatin)	2	2.55	6.6E-05	1419202_at

Gene Symbol	Gene Name	Chr	Tg-Kif1c vs NLC logFC	Tg-Kif1c vs NLC *P*-Value	Probe set ID
Ctla2a	cytotoxic T lymphocyte-associated protein 2 alpha	13	2.76	5.1E-05	1416811_s_at
Ctla2b	cytotoxic T lymphocyte-associated protein 2 beta	13	2.14	4.6E-04	1452352_at
Ctnnd1	catenin (cadherin associated protein), delta 1	2	-1.59	1.7E-04	1422450_at
Cyp27a1	cytochrome P450, family 27, subfamily a, polypeptide 1	1	-1.73	5.0E-04	1417590_at
D16Ertd472e	DNA segment, Chr 16, ERATO Doi 472, expressed	16	2.56	1.8E-04	1424724_a_at
Dnajb4	DnaJ (Hsp40) homolog, subfamily B, member 4	3	2.58	1.3E-04	1431734_a_at
Dock1	dedicator of cytokinesis 1	7	-1.85	7.0E-05	1452220_at
Dpp4	dipeptidylpeptidase 4	2	-1.84	2.8E-04	1416697_at
Dusp10	dual specificity phosphatase 10	1	2.86	1.4E-04	1417163_at
Dusp3	dual specificity phosphatase 3	11	-2.29	2.2E-05	1434472_at
Egr2	early growth response 2	10	1.37	4.7E-04	1427682_a_at
Elovl7	ELOVL family member 7, elongation of long chain fatty acids	13	1.99	1.5E-04	1424097_at
Emb	embigin	13	-1.55	1.4E-04	1415856_at
Eomes	eomesodermin homolog	9	3.72	8.7E-05	1435172_at
F13a1	coagulation factor XIII, A1 subunit	13	-2.18	2.6E-05	1448929_at
F2r	coagulation factor II (thrombin) receptor	13	3.53	3.8E-06	1450852_s_at
Fasl	Fas ligand (TNF superfamily, member 6)	1	4.44	8.6E-07	1449235_at
Fbxo18	F-box protein 18	2	-1.40	4.3E-04	1452153_at
Fbxo8	F-box protein 8	8	1.46	2.5E-04	1418510_s_at
Fcgr1	Fc receptor, IgG, high affinity I	3	-2.14	3.2E-04	1417876_at
Fdft1	farnesyl diphosphate farnesyl transferase 1	14	-1.87	1.7E-04	1438322_x_at
Fgl2	fibrinogen-like protein 2	5	1.37	2.8E-04	1421855_at
Flt1	FMS-like tyrosine kinase 1	5	-1.49	4.5E-04	1451756_at
Fn1	fibronectin 1	1	-2.17	5.8E-05	1426642_at
Fyn	Fyn proto-oncogene	10	2.64	2.1E-05	1448765_at
Gadd45b	growth arrest and DNA-damage-inducible 45 beta	10	2.15	1.7E-04	1449773_s_at
Gem	GTP binding protein	4	4.93	7.7E-07	1426063_a_at
Gimap4	GTPase, IMAP family member 4	6	2.31	4.1E-04	1424374_at
Gimap6	GTPase, IMAP family member 6	6	2.51	6.1E-05	1427891_at
Gm2a	GM2 ganglioside activator protein	11	-1.51	2.4E-04	1448241_at
Golph3l	golgi phosphoprotein 3-like	3	-1.87	3.6E-04	1425173_s_at
Gpr35	G protein-coupled receptor 35	1	-1.39	2.6E-04	1449976_a_at
Gzma	granzyme A	13	2.44	1.2E-05	1417898_a_at
Gzmb	granzyme B	14	4.18	2.4E-06	1419060_at

**Gene Symbol**	**Gene Name**	**Chr**	**Tg-Kif1c vs NLC logFC**	**Tg-Kif1c vs NLC *P*-Value**	**Probe set ID**
H28	histocompatibility 28	3	-1.92	1.1E-04	1425917_at
H2-Aa	histocompatibility 2, class II antigen A, alpha	17	-2.43	1.5E-04	1452431_s_at
H2-Ab1	histocompatibility 2, class II antigen A, beta 1	17	-2.05	3.7E-04	1451721_a_at
H2-DMa	histocompatibility 2, class II, locus DMa	17	-2.26	1.6E-04	1422527_at
H2-DMb1	histocompatibility 2, class II, locus Mb1	17	-1.64	1.3E-04	1449580_s_at
H2-Ea-ps	histocompatibility 2, class II antigen E alpha, pseudogene	17	-2.86	7.7E-06	1422892_s_at
H2-Eb1	histocompatibility 2, class II antigen E beta	17	-2.09	6.5E-05	1417025_at
H2-Gs10	MHC class I like protein GS10	17	1.89	4.7E-04	1451644_a_at
Haus3	HAUS augmin-like complex, subunit 3	5	1.66	2.3E-04	1423919_at
Hbb-b1	hemoglobin, beta adult major chain	7	3.41	2.7E-04	1417184_s_at
Hcls1	hematopoietic cell specific Lyn substrate 1	16	-1.35	4.2E-04	1418842_at
Hspa1a	heat shock protein 1A	17	-1.73	1.5E-04	1452388_at
Hspa1b	heat shock protein 1B	17	-2.82	7.9E-05	1452318_a_at
Hspa4	heat shock protein 4	11	-1.80	2.2E-04	1435194_at
Ifi202b	interferon activated gene 202B	1	-2.04	4.5E-04	1421551_s_at
Ifi204	interferon activated gene 204	1	-1.42	3.8E-04	1419603_at
Ifi27l2a	interferon, alpha-inducible protein 27 like 2A	12	-1.81	9.4E-05	1426278_at
Ifi30	interferon gamma inducible protein 30	8	-2.46	8.9E-06	1422476_at
Ifi44	interferon-induced protein 44	3	-1.57	1.4E-04	1423555_a_at
Ifitm1	interferon induced transmembrane protein 1	7	-1.66	1.6E-04	1424254_at
Ifng	interferon gamma	10	3.30	5.4E-05	1425947_at
Il13ra1	interleukin 13 receptor, alpha 1	X	-1.88	4.8E-04	1427164_at
Il18rap	interleukin 18 receptor accessory protein	1	1.57	4.2E-04	1421291_at
Il2rb	interleukin 2 receptor, beta chain	15	2.69	1.4E-04	1448759_at
Il6ra	interleukin 6 receptor, alpha	3	-1.38	4.4E-04	1452416_at
Impact	imprinted and ancient	18	1.75	2.2E-04	1415911_at
Irf4	interferon regulatory factor 4	13	-2.21	3.2E-05	1421173_at
Irg1	immunoresponsive gene 1	14	1.40	2.5E-04	1427381_at
Klf9	Kruppel-like factor 9	19	1.48	2.1E-04	1428288_at
Klra7	killer cell lectin-like receptor, subfamily A, member 7	6	3.17	9.0E-06	1451664_x_at
Klrd1	killer cell lectin-like receptor, subfamily D, member 1	6	2.76	5.4E-05	1460245_at
Klrg1	killer cell lectin-like receptor subfamily G, member 1	6	3.29	5.1E-06	1420788_at
Klrk1	killer cell lectin-like receptor subfamily K, member 1	6	2.63	3.3E-05	1450495_a_at
Kpna1	karyopherin (importin) alpha 1	16	1.68	4.7E-04	1449505_at

Gene Symbol	Gene Name	Chr	Tg-Kif1c vs NLC logFC	Tg-Kif1c vs NLC *P*-Value	Probe set ID
Lrrc33	leucine rich repeat containing 33	16	-1.27	4.6E-04	1451174_at
Ly86	lymphocyte antigen 86	13	-1.71	2.4E-04	1422903_at
Lyl1	lymphoblastomic leukemia 1	8	-2.05	2.8E-04	1419120_at
Marveld1	MARVEL (membrane-associating) domain containing 1	19	-1.36	3.2E-04	1439381_x_at
Mpeg1	macrophage expressed gene 1	19	-1.71	1.6E-04	1427076_at
Ms4a4b	membrane-spanning 4-domains, subfamily A, member 4B	19	2.87	1.0E-05	1423467_at
Ms4a6c	membrane-spanning 4-domains, subfamily A, member 6C	19	-1.97	3.7E-05	1450234_at
Ms4a6d	membrane-spanning 4-domains, subfamily A, member 6D	19	-1.52	4.5E-04	1419599_s_at
Msr1	macrophage scavenger receptor 1	8	-1.97	1.2E-04	1448061_at
Myadm	myeloid-associated differentiation marker	7	-1.44	4.2E-04	1439389_s_at
Naip5	NLR family, apoptosis inhibitory protein 5	13	-1.39	4.0E-04	1421525_a_at
Ndufv1	NADH dehydrogenase (ubiquinone) flavoprotein 1	19	-1.50	3.7E-04	1415966_a_at
Nfam1	Nfat activating molecule with ITAM motif 1	15	-1.37	5.0E-04	1428790_at
Nkg7	natural killer cell group 7 sequence	7	2.72	8.5E-05	1450753_at
Nr1d2	nuclear receptor subfamily 1, group D, member 2	14	2.81	9.7E-06	1416958_at
Nr4a2	nuclear receptor subfamily 4, group A, member 2	2	1.83	2.4E-04	1450749_a_at
Nrarp	Notch-regulated ankyrin repeat protein	2	2.77	1.7E-04	1417985_at
P2rx4	purinergic receptor P2X, ligand-gated ion channel 4	5	-2.05	2.7E-04	1425525_a_at
Paip2b	poly(A) binding protein interacting protein 2B	6	2.13	3.7E-05	1451125_at
Pfkp	phosphofructokinase, platelet	13	1.83	6.0E-05	1416069_at
Pigp	phosphatidylinositol glycan anchor biosynthesis, class P	16	1.44	3.5E-04	1436038_a_at
Pik3r1	phosphatidylinositol 3-kinase, regulatory subunit, polypeptide 1	13	2.58	2.3E-05	1425514_at
Pitpnc1	phosphatidylinositol transfer protein, cytoplasmic 1	11	1.64	4.8E-04	1435066_at
Pld4	phospholipase D family, member 4	12	-2.19	7.4E-05	1433678_at
Plek	pleckstrin	11	1.79	3.6E-04	1448748_at
Pltp	phospholipid transfer protein	2	-1.70	1.3E-04	1456424_s_at
Prf1	perforin 1 (pore forming protein)	10	3.01	7.0E-05	1451862_a_at
Pros1	protein S (alpha)	16	-1.71	1.3E-04	1426246_at
Ptger4	prostaglandin E receptor 4 (subtype EP4)	15	1.39	4.3E-04	1424208_at
Ptpro	protein tyrosine phosphatase, receptor type, O	6	-2.20	2.1E-05	1417676_a_at
Rgs1	regulator of G-protein signaling 1	1	2.91	4.9E-05	1417601_at
Rgs10	regulator of G-protein signalling 10	7	-2.28	4.7E-05	1416882_at
Rnase4	ribonuclease, RNase A family 4	14	-1.95	9.8E-05	1422603_at
Rras	Harvey rat sarcoma oncogene, subgroup R	7	-2.05	2.6E-05	1418448_at

Gene Symbol	Gene Name	Chr	Tg-Kif1c vs NLC logFC	Tg-Kif1c vs NLC *P*-Value	Probe set ID
Rras2	related RAS viral (r-ras) oncogene homolog 2	7	1.30	4.9E-04	1417398_at
S100a4	S100 calcium binding protein A4	3	-1.84	1.6E-04	1424542_at
Samhd1	SAM domain and HD domain, 1	2	-1.37	3.3E-04	1434438_at
Scarb1	scavenger receptor class B, member 1	5	-2.73	2.2E-05	1455820_x_at
Serpinb9	serine (or cysteine) peptidase inhibitor, clade B, member 9	13	3.72	3.8E-05	1422601_at
Slain1	SLAIN motif family, member 1	14	1.85	1.2E-04	1424824_at
Slc16a1	solute carrier family 16, member 1	3	1.58	1.8E-04	1415802_at
Slc41a2	solute carrier family 41, member 2	10	2.44	1.5E-05	1452445_at
Spata13	spermatogenesis associated 13	14	1.82	1.3E-04	1454656_at
Spry2	sprouty homolog 2 (Drosophila)	14	3.53	4.6E-06	1436584_at
St3gal6	ST3 beta-galactoside alpha-2,3-sialyltransferase 6	16	2.28	3.4E-04	1449078_at
Stat4	signal transducer and activator of transcription 4	1	2.68	1.9E-04	1448713_at
Stom	stomatin	2	-1.71	1.5E-04	1438910_a_at
Tbx21	T-box 21	11	2.40	4.5E-04	1449361_at
Tgfbi	transforming growth factor, beta induced	13	-2.74	1.1E-05	1415871_at
Tmem176a	transmembrane protein 176A	6	-3.01	2.1E-04	1423909_at
Tmem176b	transmembrane protein 176B	6	-2.52	8.1E-06	1418004_a_at
Tmtc4	transmembrane and tetratricopeptide repeat containing 4	14	1.62	1.4E-04	1428113_at
Tnfrsf13b	tumor necrosis factor receptor superfamily, member 13b	11	-2.04	1.4E-04	1423182_at
Tnfsf12	tumor necrosis factor (ligand) superfamily, member 12	11	-1.68	2.1E-04	1418345_at
Tppp3	tubulin polymerization-promoting protein family member 3	8	-2.10	1.8E-04	1416713_at
Tspan31	tetraspanin 31	10	-1.84	2.3E-04	1416556_at
Tulp4	tubby like protein 4	17	2.61	5.2E-04	1448548_at
Ugcg	UDP-glucose ceramide glucosyltransferase	4	2.59	2.5E-04	1421268_at
Usp18	Ubiquitin-specific peptidase 18	NA	-2.08	7.1E-05	1418191_at
Vps37c	vacuolar protein sorting 37C	19	-1.85	1.5E-04	1426227_s_at
Vwa5a	von Willebrand factor A domain containing 5A	9	-2.27	2.8E-04	1426221_at
Xist	inactive X specific transcripts	X	1.97	1.4E-04	1427262_at
2310044G17Rik	RIKEN cDNA 2310044G17 gene	12	1.76	1.3E-04	1424915_s_at
4930453N24Rik	RIKEN cDNA 4930453N24 gene	16	1.56	1.8E-04	1423976_at
