# Supplementary material for: Identification of Orch3, a Locus Controlling Dominant Resistance to Autoimmune Orchitis, as Kinesin Family Member 1C
Source: PLoS Genet. 2012 Dec 27;8(12):e1003140. doi: 10.1371/journal.pgen.1003140 (PMC3531464; doi:10.1371/journal.pgen.1003140)
Supplement: Table S2 — Transgenic expression of Kif1cD2 on CD11b+ cells influences pathways involving MHC Class II (genes in bold). TCRβ−IA/IE+CD11c−CD11b+ myeloid cells were harvested from naïve NLC and Tg-Kif1cD2 mice and differential gene expression was detected using Affymetrix GeneChip Mouse Genome 430A 2.0 Arrays. 1Pathway analysis was conducted using Ingenuity Pathway Analysis software (Ingenuity Systems, www.ingenuity.com). To ensure biological relevance, cell type was restricted to B-cells, dendritic cells, and macrophages. (PDF) [file pgen.1003140.s005.pdf]

**Ingenuity Canonical Pathways**[1]	***P*-value**	**Molecules**
T Helper Cell Differentiation	2.88E-10	Il6st,**H2-Dma**,Ifng,Il2rg,Il6r,**H2-Aa**,**H2-Eb1**,**H2-Dmb1**,**H2-Ab1**,Bcl6,Tbx21,Il18r1,Stat4,Cd28,Cd40,**H2-Ea-Ps**,Il10ra,Gata3,Stat1
Autoimmune Thyroid Disease Signaling	1.38E-08	**H2-Dma**,**H2-Aa**,**H2-Eb1**,**H2-Dmb1**,**H2-Ab1**,Cd28,Prf1,Cd40,**H2-Ea-Ps**,**H2-Gs10**,Faslg,Gzmb
Allograft Rejection Signaling	1.95E-08	Ifng,**H2-Dma**,**H2-Aa**,**H2-Eb1**,**H2-Dmb1**,**H2-Ab1**,Cd28,Prf1,Cd40,**H2-Ea-Ps**,**H2-Gs10**,Faslg,Gzmb
Graft-versus-Host Disease Signaling	3.02E-08	Ifng,**H2-Dma**,**H2-Aa**,**H2-Eb1**,**H2-Dmb1**,**H2-Ab1**,Cd28,Prf1,**H2-Ea-Ps**,**H2-Gs10**,Faslg,Gzmb
Cytotoxic T Lymphocyte-mediated Apoptosis of Target Cells	1.70E-07	Cd247,**H2-Dma**,**H2-Aa**,**H2-Eb1**,**H2-Dmb1**,**H2-Ab1**,Bcl2,Prf1,**H2-Ea-Ps**,**H2-Gs10**,Faslg,Gzmb
Crosstalk between Dendritic Cells and Natural Killer Cells	4.17E-07	Ifng,Il2rg,Klrd1,**H2-Eb1**,Ltb,Cd83,Il2rb,Cd28,Tlr4,Prf1,Cd40,Klrc4-Klrk1/Klrk1,**H2-Ea-Ps**,**H2-Gs10**,Tlr7,Faslg
Type I Diabetes Mellitus Signaling	9.33E-07	Cd247,**H2-Dma**,Ifng,Jak1,Myd88,**H2-Aa**,**H2-Eb1**,**H2-Dmb1**,**H2-Ab1**,Bcl2,Cd28,Prf1,Nfkbia,**H2-Ea-Ps**,**H2-Gs10**,Stat1,Faslg,Gzmb
Communication between Innate and Adaptive Immune Cells	3.09E-06	Ifng,**H2-Eb1**,Cd83,Ccl5,Ccl9,Cd28,Tlr4,Ccl4,Cd40,Tnfsf13,**H2-Ea-Ps**,**H2-Gs10**,Tlr7,Ccl3l1/Ccl3l3,Tnfrsf13b
CTLA4 Signaling in Cytotoxic T Lymphocytes	8.71E-06	Cd247,**H2-Dma**,Fyn,Ppp2r2a,Pik3r1,**H2-Aa**,**H2-Eb1**,**H2-Dmb1**,**H2-Ab1**,Ap2a2,Ap2s1,Cd28,Lck,**H2-Ea-Ps**,Ptpn22
Altered T Cell and B Cell Signaling in Rheumatoid Arthritis	8.71E-06	Ifng,**H2-Dma**,**H2-Aa**,**H2-Eb1**,Ltb,**H2-Dmb1**,**H2-Ab1**,Cd28,Tlr4,Cd40,Tnfsf13,**H2-Ea-Ps**,Tlr7,Tnfrsf13b,Faslg
Calcium-induced T Lymphocyte Apoptosis	1.23E-05	Cd247,**H2-Dma**,Lck,Prkcd,**H2-Ea-Ps**,**H2-Aa**,Nr4a1,**H2-Eb1**,**H2-Dmb1**,Ppp3cc,**H2-Ab1**,Prkca
Nur77 Signaling in T Lymphocytes	2.40E-05	Cd247,**H2-Dma**,Cd28,**H2-Ea-Ps**,**H2-Aa**,Nr4a1,**H2-Eb1**,**H2-Dmb1**,Ppp3cc,**H2-Ab1**,Bcl2
OX40 Signaling Pathway	2.40E-05	Cd247,**H2-Dma**,Nfkbia,**H2-Ea-Ps**,**H2-Aa**,**H2-Gs10**,**H2-Eb1**,**H2-Dmb1**,**H2-Ab1**,Bcl2
iCOS-iCOSL Signaling in T Helper Cells	2.82E-05	Cd247,**H2-Dma**,Il2rg,Bad,Pik3r1,**H2-Aa**,**H2-Eb1**,**H2-Dmb1**,Ppp3cc,**H2-Ab1**,Il2rb,Cd28,Lck,Nfkbia,Cd40,**H2-Ea-Ps**
Hepatic Fibrosis / Hepatic Stellate Cell Activation	3.16E-05	Ifng,Il18rap,Ccr5,Fn1,Il1rapl2,Flt1,Il6r,Smad7,Ccl5,Bcl2,Il1r2,Tlr4,Ly96,Cd4,Pdgfra,Il10ra,Stat1,Faslg
Antigen Presentation Pathway	5.62E-05	Ifng,**H2-Dma**,**H2-Ea-Ps**,**H2-Aa**,**H2-Gs10**,**H2-Eb1**,**H2-Dmb1**,Cd74
IL-4 Signaling	5.75E-05	**H2-Dma**,Il2rg,Jak1,Irf4,Il13ra1,Rras,Pik3r1,**H2-Aa**,**H2-Eb1**,**H2-Dmb1**,**H2-Ab1**,Rras2,**H2-Ea-Ps**
Dendritic Cell Maturation	5.75E-05	**H2-Dma**,Myd88,Pik3r1,Mapk3,**H2-Aa**,**H2-Eb1**,Ltb,**H2-Dmb1**,Cd83,**H2-Ab1**,Fcgr1a,Stat4,Tlr4,Nfkbia,Cd40,**H2-Ea-Ps**,**H2-Gs10**,Stat2,Irf8,Stat1
PKCθ Signaling in T Lymphocytes	1.17E-04	Cd247,**H2-Dma**,Fyn,Rras,Mapk3,Pik3r1,**H2-Aa**,**H2-Eb1**,**H2-Dmb1**,Ppp3cc,**H2-Ab1**,Cd28,Lck,Rras2,Nfkbia,**H2-Ea-Ps**
Interferon Signaling	1.78E-04	Ifng,Oas1,Jak1,Ptpn2,Ifitm1,Stat2,Stat1,Bcl2
